# Road Infrastructure and Primate Conservation: Introducing the Global Primate Roadkill Database

**DOI:** 10.3390/ani13101692

**Published:** 2023-05-19

**Authors:** Laura C. Praill, Timothy M. Eppley, Sam Shanee, Pamela M. K. Cunneyworth, Fernanda D. Abra, Néstor Allgas, Hassan Al-Razi, Marco Campera, Susan M. Cheyne, Wendy Collinson, Giuseppe Donati, Birthe Linden, Sophie Manson, Marjan Maria, Thais Q. Morcatty, K. A. I. Nekaris, Luciana I. Oklander, Vincent Nijman, Magdalena S. Svensson

**Affiliations:** 1 Faculty of Humanities and Social Sciences, Oxford Brookes University, Oxford OX3 0BP, UK; 2 Pandrillus Cameroon, Limbe Wildlife Centre, Limbe P.O. Box 878, Cameroon; 3 Wildlife Madagascar, Antananarivo 101, Madagascar; 4 Department of Anthropology, Portland State University, Portland, OR 97201, USA; 5 Conservation Science and Wildlife Health, San Diego Zoo Wildlife Alliance, Escondido, CA 92027, USA; 6 Asociación Neotropical Primate Conservation Perú, Moyobamba 22001, Peru; 7 Neotropical Primate Conservation, Cornwall PL11 3JQ, UK; 8 Colobus Conservation, P.O. Box 5380, Diani 80401, Kenya; 9 Smithsonian National Zoo and Conservation Biology Institute—Center for Conservation and Sustainability, Washington, DC 20560, USA; 10 ViaFAUNA Estudos Ambientais, São Paulo 04125-120, SP, Brazil; 11 Instituto Pró-Carnívoros, Atibaia 12945-010, SP, Brazil; 12 Bangladesh Slow Loris Research and Conservation Project, 531/2, Shahidbagh, Dhaka 1217, Bangladesh; 13 Faculty of Life Sciences, Oxford Brookes University, Oxford OX3 0BP, UK; 14 Faculty of Science, Engineering and Agriculture, University of Venda, Thohoyandou 0950, South Africa; 15 The Endangered Wildlife Trust, Wierda Park 0149, South Africa; 16 Lajuma Research Centre, Louis Trichardt 0920, South Africa; 17 Little Fireface Project, Chipaganti, Cisurupan, Garut 44163, Indonesia; 18 Grupo de Investigación en Genética Aplicada, UNAM-CONICET, Posadas N3304, Argentina; 19 Neotropical Primate Conservation Argentina, Puerto Iguazú N3370, Argentina

**Keywords:** anthropogenic impact, citizen science, conservation, primates, road ecology, wildlife mortalities, wildlife-vehicle collisions

## Abstract

**Simple Summary:**

When wildlife cross roads, they risk fatality due to collisions with automobiles and motorbikes. As road infrastructure networks rapidly expand globally, especially in the tropics and subtropics, primates are increasingly at risk from these collisions. We created the Global Primate Roadkill Database (GPRD) as a comprehensive standardized repository to document incidents of primates killed by vehicular collisions. For each primate roadkill event, we recorded the species, location, and the year and month the incident was observed. As of February 2023, we collated over 2800 roadkill incidents, involving at least 107 primate species, from 41 countries. The lack of data from a large number of primate range countries did not necessarily indicate that primate roadkill events do not occur there, but more likely reflects underreporting. Given the value of these data for addressing both local and global research questions, we encourage conservationists and citizen scientists to contribute to the GPRD so that, together, we can better understand the impact road infrastructure has on primates.

**Abstract:**

As road infrastructure networks rapidly expand globally, especially in the tropics, previously continuous habitats are being fragmented, resulting in more frequent wildlife–vehicle collisions (WVC). Primates are widespread throughout many sub-/tropical countries, and as their habitats are fragmented, they are increasingly at risk of WVC. We created the Global Primate Roadkill Database (GPRD), the largest available standardized database of primate roadkill incidents. We obtained data from published papers, un-published and citizen science databases, anecdotal reports, news reports, and social media posts. Here, we describe the collection methods for the GPRD and present the most up-to-date version of the database in full. For each primate roadkill incident, we recorded the species killed, the exact location, and the year and month the roadkill was observed. At the time of publication, the GPRD includes 2862 individual primate roadkill records from 41 countries. As primates range in more than twice as many countries, the absence of data from these countries is not necessarily indicative of a lack of primate vehicular collisions. Given the value of these data for addressing both local and global research questions, we encourage conservationists and citizen scientists to contribute to the GPRD so that, together, we can better understand the impact road infrastructure has on primates and evaluate measures which may help mitigate risk-prone areas or species.

## 1. Introduction

Roads provide important infrastructure within both local and global economies, and can benefit and improve the quality of life for impoverished human populations that were previously limited in what they could access. Simultaneously, roads can also be problematic for biodiversity conservation in several respects. For example, roads can decrease the quality of the surrounding habitat by reducing its size, decreasing connectivity between fragments and increasing the length of the edge of habitats. Thus, road development may adversely affect wildlife populations by reducing available suitable habitat, limiting species movement, and disrupting processes such as gene flow and dispersal, e.g., [1,2,3,4]. The expansion of the human population led to the rapid growth of linear infrastructure (e.g., road) networks, as well as increased traffic volume, in previously undeveloped countries throughout the tropics, threatening local wildlife populations [5]. An increase in industrial-level agriculture, cattle ranching, natural resource extraction (e.g., mining and logging), and even the building of large hydroelectric projects and power line corridors, requires the construction of massive transportation networks (i.e., road and railways). Such road building triggers deforestation and fragmentation, and often spawns networks of secondary and tertiary roads that greatly disrupt natural habitats in previously isolated areas [6]. Predictions suggest that 25 million km of newly paved roads will be built by 2050; thus, it is growing increasingly imperative to understand which species are impacted by road traffic collisions, where they occur, to what extent and why [7].

Non-human primates (hereafter primates) inhabit most tropical and sub-tropical regions in the Neotropics, Africa, and Asia, where they play an important role in the functioning of these ecosystems [6]. Within their range countries, primates face a variety of global and local pressures, including anthropogenic activities such as land conversion for agricultural and human population expansion, led to an alarming decline in their population sizes [6,8]. Of the 522 primate species listed on the International Union for Conservation (IUCN) Red List, as of February 2023, 66.7% are categorized as threatened (i.e., Vulnerable, Endangered or Critically Endangered) species, and 89.3% have a declining population trend [9].

Moreover, according to the IUCN Threats Classification Scheme (Version 3.2), 19.4% primate species are listed as threatened by roads and railroads [9]. Road networks are expanding rapidly in and around primate habitats, including remote forests that act as strongholds for some species [10,11,12,13]. As a result, wildlife-vehicle collisions (WVCs) involving primates are becoming more frequent [14]. Despite many primates being arboreal, roads present potential obstacles that often force primates to descend to the ground to access fragmented forest patches on the other side [15]. Within both urban and rural areas, primates may frequently cross roads for access to resources and/or for reproductive opportunities, leading to WVCs and potential death [16,17].

While roads may appear as a small risk to global primate populations when compared to other risk factors, it is an understudied threat that is known to negatively impact wildlife persistence in local hotspots [18]. Therefore, the collation of primate roadkill data may indicate the true extent of the threat that WVCs pose to their populations. In fact, primate mortality along roads is likely underestimated due to the absence of systematic roadkill monitoring in most primate range countries [19], although some roadkill studies include primates in their published datasets, e.g., [15,20,21,22]. Of the few primate-focused roadkill studies, Hetman and colleagues [23] identified several globally threatened primate species involved in fatal WVCs, including a high incidence of infant/juvenile mortality. This supports the idea that WVCs impact on at-risk species and vulnerable individuals is widespread.

Despite the dearth of standardized data, developing a comprehensive understanding of how primate roadkill affects populations is essential to inform conservationists of WVC hotspots. For at-risk species, any fatality within a population can have damaging effects, including reduced gene flow, population decline, and local extinction [24]. By continuously collating and analyzing primate roadkill data, we can address and evaluate the impact that WVCs have on primate populations, and more effectively identify and implement species/regional-tailored mitigation strategies.

The use of anecdotal data within primatology declined over the years [25]; however, it is useful for documenting and describing purportedly rare incidents such as primate roadkill and expanding our knowledge of underrepresented taxa and sites [26,27]. In response to the growing yet anecdotal nature of primate roadkill observations, we introduce the Global Primate Roadkill Database (GPRD). This open access online data repository will act as a comprehensive resource for anyone studying this issue, and we will regularly update the standardized database with validated records contributors. Sharing biodiversity data, and providing centralized databases, is important to enable decision makers to effectively work towards global conservation goals, and, thus, we decided the GPRD will be globally accessible and free [28,29]. Below, we detail the creation of the GPRD, provide a synthesis of the current data, and discuss the observed general trends in primate WVCs.

## 2. Materials and Methods

Commencing on 26 May 2020, we circulated a Google Forms questionnaire to collect anecdotal data on primate roadkill and collate it into a standardized database. Participants were contacted through a variety of means: relevant Facebook groups (e.g., Fragmentation-related Road/Railway & Electrocution Wildlife Casualties (Asia), the IUCN/SSC Primate Specialist Group, Section for Human-Primate Interactions); broader-scope Facebook groups (e.g., Primate Society of Great Britain); directly emailing researchers known to publish on related/relevant topics; contacting relevant projects (e.g., the Langur Project Penang); asking participants to forward surveys to colleagues; and by distributing the survey on other social media sites such as Twitter, Instagram, and LinkedIn.

Ethical clearance was obtained from the School of Social Sciences, Oxford Brookes University, prior to commencing the data collection. Participants had the option to be anonymous, or to share their contact details in the case they were willing to be contacted for additional information.

Additionally, we used Google Scholar to conduct a systematic search of published research for primate roadkill data. Keywords used were “ape”, “monkey”, “primate”, and scientific and common names of primate species, as well as “roadkill”, “road kill”, “road mortality” and “vehicular mortality”. These phrases were repeated using the most prominent languages (e.g., Spanish, French, and Indonesian) from primate range countries. All examples of primate roadkill were included. Using the same keywords, we also searched for reports about primate roadkill on social media (e.g., Facebook, Twitter, YouTube) and on Google News. We also included data from previous, including some more localized, databases with primate roadkill records. This included both publicly available databases, e.g., Global Biodiversity Information Facility [30] and iNaturalist, as well as databases donated by organizations. There was no restriction placed on the age of the data, which allows us to analyze primate roadkill trends over time.

In March 2023, we identified several geographical ‘coldspots’, i.e., countries with primates but from where we had yet to record any primate roadkill. We then specifically searched for records in these countries using each country’s majority languages. The languages included were Portuguese (for Angola, Cape Verde, Guinea-Bissau, Equatorial Guinea, Mozambique, East Timor, and São Tomé and Príncipe), Dutch (for Suriname and St Maarten), French (for Morocco, Senegal, Côte d’Ivoire, Cameroon, and Gabon), and Malay/Indonesian (for Brunei and East Timor).

For primate species identification, we followed the taxonomy of The Mammals of the World Volume 3 Primates [31], and updates where relevant. We included reported incidents in the Global Primate Roadkill Database only when primates were identified to at least the genus level. Most primates were identified to species level, some to the subspecies level. Submissions from non-experts (e.g., citizen scientists in social media groups such as ROAD WATCH: Indian Wildlife Roadkill Monitoring Network Group on Facebook or roadkill data collection apps) were included so long as the reliability of the species identification validated via photograph(s) by an expert. We only included primate roadkill records when it was evident that the primate in question had indeed died upon impact with a vehicle or shortly thereafter due to the collision. Cases where a primate was hit by a vehicle or a motorbike but where there was no evidence of it dying afterwards as a result were excluded. Likewise, cases where primates were hit by a vehicle but were merely injured were also excluded [32].

We focused on primates in primate range countries, historical primate range countries, or territories with long-term free-ranging populations. Thus, Hong Kong historically was within the range of the rhesus macaque (*Macaca mulatta*), but the indigenous population became extinct around the 1960s [33]. The macaques that are found in Hong Kong at present are the descendants of various species of macaque, including rhesus macaques, long-tailed macaques (*M. fascicularis*), and Japanese macaques (*M. fuscata*), that were released in the territory in the first half of the 20th century. Several of these species have hybridized and hence the present population comprises hybrids and possibly non-native species in addition to the native rhesus macaque; all are included in the GPRD. Green monkeys (*Chlorocebus sabaeus*) and vervet monkey (*C. pygerythrus*) are native to Africa, but were introduced to several Caribbean islands from the mid-1600s onwards, and established self-sustaining populations on Barbados, St. Kitts, Nevis, and Sint Maarten [34,35,36], as well as in the Cape Verde Islands off the west coast of Africa [37]. Any primate roadkill records from these islands were included in the database.

For reports that indicated ‘several’ or ‘a few’ individuals, we conservatively recorded this as three in the GPRD. We included the geographic coordinates (decimal degrees) from the reports, converting them when necessary. If coordinates were unavailable, we used the information in the report (e.g., name of road, kilometer number on road, village) to obtain the approximate location along the road. In cases where only a broad area was specified (e.g., district or protected area), we used coordinates for the center of the district or protected area. Due to the variety of sources from which data were obtained, care was taken to review all points for duplicates and errors.

In creating our database, we were inspired by other freely available databases that were created as a result of collaborative efforts by often large numbers of research teams, including the PREDICTS (Projecting Responses of Ecological Diversity In Changing Terrestrial Systems) database, GrassPlot (a database of scale-dependent phytodiversity patterns in Palaearctic grasslands), TRY (a global database of plant traits) and the national assessment of wildlife mortality in Ecuador [38,39,40,41,42]. Our database (GPRD) includes, where available, the following information for each entry: the data source (e.g., database, anecdote, research paper, social media, or donated private data), the primate species’ scientific and common names and Red List status, the year and month of the incident observation, the location (e.g., country, state, district, National Park, town/city, road), GIS coordinates (i.e., latitude and longitude), demographic details (e.g., number of individuals killed, including the age and sex). The database is an open resource for researchers and decision makers to use freely to aid scientific discovery and conservation efforts. The database will be updated and maintained by the lead author by accepting new submissions following the methods described here.

**Figure 1:**
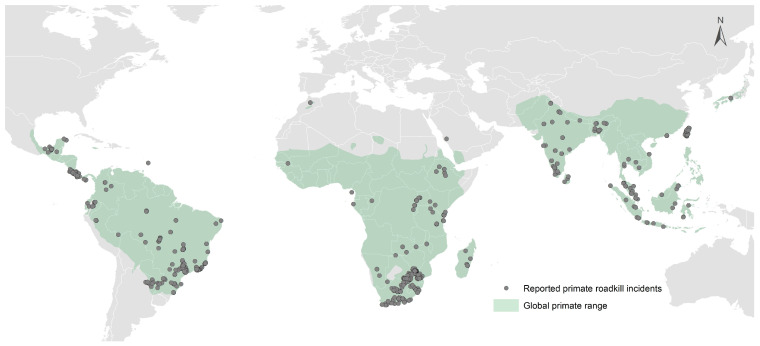
Geographical locations of all primate roadkill incidents included in the Global Primate Roadkill Database as of May 2023.

To visualize the entries in the GPRD, we created a map in ArcGIS (10.8.2) displaying the incidents of primate roadkill along with global extent primate ranges (Figure 1). Most GPRD entries include a date (month and year), although for some entries this is presented as a range (years). For those entries that contained data on the year the incident occurred, we tallied the number of incidents and the number of individual primates killed, and present that as to illustrate the accumulation of data in the database.

## 3. Results

**Figure 2:**
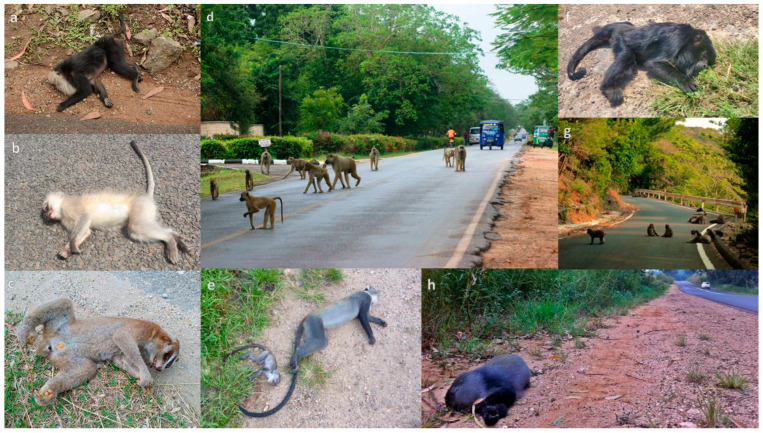
Primates on roads and primate roadkill: (**a**) lion-tailed macaque (*Macaca silenus*), India (© P. Jeganathan, CC-BY); (**b**) vervet monkey (*Chlorocebus pygerythrus*), South Africa (© B. Linden); (**c**) greater slow loris (*Nycticebus coucang*), Malaysia (© N. Yamaguchi); (**d**) yellow baboon (*Papio cynocephalus*), Kenya (© A. Donaldson); (**e**) adult female and infant Zanzibar Sykes’ monkey (*Cercopithecus mitis albogularis*), Kenya (© A. Donaldson); (**f**) black and gold howler monkey (*Alouatta caraya*), Argentina (© L. Oklander); (**g**) Japanese macaques (*M. fuscata*), Japan (© M. Mueller, CC-BY); (**h**) samango monkey (*Cercopithecus mitis*), South Africa (© B. Linden).

As of February 2023, the Global Primate Roadkill Database (GPRD) recorded incidents from 41 countries (Figure 1), across at least 107 species (Table 1, Figure 2). In total, 2862 individual primates were reported as roadkill globally between 1987 and 2023. Out of all roadkill incidents reported, 62.7% came from databases, 24.1% from published papers, 6.0% from theses, 5.7% were anecdotal observations (i.e., reported through our questionnaire or observed by one of the authors), and 1.4% were reported on social media pages or other online news forums. The five genera with the most reported roadkill incidents were *Callithrix* (668 individuals), *Cercopithecus* (591), *Chlorocebus* (377), *Macaca* (324), *Colobus* (240), and *Papio* (143). The species with the most reported roadkill incidents were Zanzibar Sykes’ monkeys (*Cercopithecus mitis albogularis*, 557 individuals), vervet monkeys (*Chlorocebus pygerythrus*, 371), and white-headed marmosets (*Callithrix geoffroyi*, 215).

The majority of primate roadkill incidences (i.e., individuals), comprising 48 species, were classified as Least Concern or Near Threatened by the IUCN Red List (Table 2). A larger number of species, but fewer individuals, were classified as globally threatened, including 16 roadkill incidents of Critically Endangered primate species.

Geographically, primate roadkill was the most reported from Africa, representing 51.9% of all cases, with the highest occurrences in Kenya and South Africa (Table 1; Figure 2).

The Neotropics represented 32.8% of reported cases, mainly from Brazil followed by Mexico and Costa Rica. The least number of incidents were reported from Asia, representing 15.2%, with most cases being reported from India and China. Of the four most primate-rich countries, i.e., Brazil, Democratic Republic of Congo (DRC), Madagascar, and Indonesia, only Brazil had a large number of primate roadkill records, whilst DRC and Madagascar stand out as having exceptionally few records.

**Figure 3:**
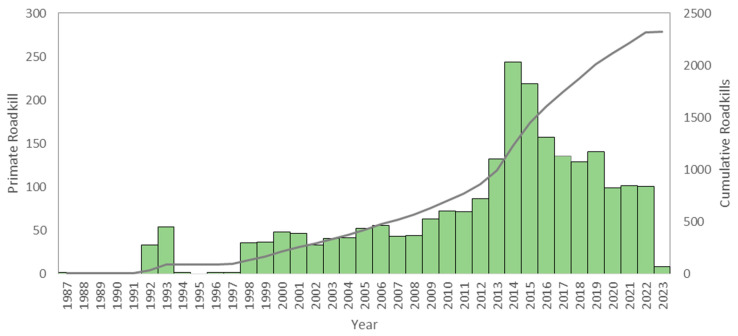
Number of primate road kills included in the Global Primate Roadkill Database based on the year of the incident (bars), showing a gradual increase in the 1990s and 2000s, and an acceleration of records in the 2010s. The secondary axis provides the cumulative number of roadkill incidents (line).

The GPRD contains data on primate roadkill from 1987 to 2023, and for 2282 of 2862 records, we have the approximate date (i.e., month and year) of the incident. Figure 3 shows the number of records by year and the cumulative number over time. Numbers were below 100 in the 1990s and 2000s before increasing to approximately 150 incidents per year in 2010s and 2020s.

## 4. Discussion

The Global Primate Roadkill Database (GPRD) was created to store and organize roadkill incidents in a standardized dataset, making it useful for future scientific analysis, and to be used by conservation practitioners and policy makers (e.g., for global, regional and national Red List assessments and conservation plans). As incidents of primate roadkill are occasionally reported in the scientific literature, they are still rare and, thus, we encourage conservationists and citizen scientists to continue to contribute to this database. The GPRD is useful to identify countries and local areas that are hotspots for primate roadkill. Collecting, evaluating, and modelling these data may be useful to prevent future WVCs and perhaps even local extinctions. Through the collection of roadkill reports across the globe, the GPRD will be useful in order to better allocate research effort and limited conservation funds to primate species that may be most affected.

One of the reasons for creating the GPRD was to provide a framework for those that have collected primate roadkill data, so that they could be added into a standardized database. It is rare for anecdotal records, and even full datasets, to be published in a standardized way; thus, collating these records provides a platform for saving these potentially valuable data from being lost [43]. In our opinion, it is imperative that the data generated become freely available to other researchers, civil engineers, policy makers, and anyone that is interested in using these data for modelling. Similarly, the creators of the PREDICTS database argued that openness of data (and with it, the reproducibility and transparency that open data can confer) offer benefits to all areas of scientific research and indeed policy. The GPRD is comparable to numerous open access data repositories, from taxonomically and geographically diverse databases such as PREDICTS [40], the global database of ant species abundances [44], and the global platform for linking soil biodiversity data [45]. There are also more regionally focused databases such as the country-specific terrestrial camera trap survey data [46], or even GrassPlot [39]. Perhaps most similar to the GPRD is TRY, a global database for plant traits [38], which aims for large geographical coverage, open infrastructure and is set-up as a ‘work in progress’ with new data being added as they becomes available. While some of the original authors analyzed parts of the data from their respective databases, the key motivation was often explicitly expressed as aiming to allow other scientists to make use of the available dataset. Like the GPRD, many of these global open databases have websites that are regularly updated.

### 4.1. Frequently Reported Primate Species

Of the top six genera with the most roadkill incidents within the GPRD, five were cercopithecines. Cercopithecines may be overrepresented in reports as they are a species-rich, geographically wide-ranging group throughout Africa and Asia, of which many species are known for their (semi) terrestrial lifestyle [31]. Some primate species adapt well to changing environments, as they are behaviorally flexible and make dietary adjustments within anthropogenic landscapes [47,48,49,50,51,52], and nearly 80% of cercopithecine species having adapted to live in disturbed habitat [53,54,55]. Despite this, over 72% of cercopithecine species are classified as at risk [9]. Their ability to successfully exploit human-modified environments, including anthropogenic food sources, may contribute to their threatened status as more frequent contact with local humans leads to conflict [51,56,57]. For example, in some areas of Tanzania where baboons (*Papio* spp.) range in human-modified landscapes, locals report negative attitudes towards baboons, to the extreme degree of reports of drivers swerving their vehicles to hit baboons [22,58]. Cercopithecines such as macaques (*Macaca* spp.), vervet monkeys (*Chlorocebus* spp.), and baboons (*Papio* spp.) are often cited as sources of human–wildlife conflict; therefore, local attitudes may make species vulnerable to persecution, whether on the road or by other means [59,60]. Roadkill of species that are generally not appreciated by local communities may be under-reported relative to those that are more accepted or that are the focus of conservation initiatives [29].

While cercopithecines are heavily represented in the GPRD, the genus with the most reported roadkill incidents were marmosets (*Callithrix* spp.), a group of small monkeys from the Neotropics. Of the six *Callithrix* species, three were categorized under Least Concern and represented the vast majority of the reported roadkill from this genus. Like most Neotropical primates, marmosets are highly arboreal, but with habitats becoming increasingly degraded and fragmented, species may be forced to cross roads and enter urban areas in search of potential food resources and reproductive opportunities [61,62]. In fact, some studies indicated that marmosets living near humans adapt their behaviors to cope within these anthropogenic environments, but this likely places them at greater risk of WVCs and death [63,64].

### 4.2. IUCN Status

Nearly 76% of primate individuals reported as roadkill in the GPRD were of species that are assessed as not being globally threatened, whilst 24.2% of the reported incidents of primate roadkill within the GPRD were of species that are considered to be at risk of extinction (Table 2). In general, primate species listed as Least Concern may have broader ranges, larger and denser populations [65], and may frequently exhibit greater ecological flexibility, including more ground use. However, this does not mean they are not vulnerable to anthropogenic pressure, species may be listed under Least Concern due to a lack of adequate data [66]. Some primate species previously listed as not threatened were recently reassessed as at risk due to shrinking natural habitat and expanding urbanization [67,68]. For example, the populations of bonnet macaques (*Macaca radiata*) in Karnataka, India, declined nearly 65% since 1989 [69]. While these declines were linked to habitat loss, WVCs may have also contributed as roads and highways greatly expanded throughout the remaining habitat of bonnet macaques [70,71]. As a result, bonnet macaques were reclassified from Least Concern to Vulnerable in 2020 [72].

The three species within the GPRD with the most reported roadkill incidents were all classified as Least Concern; however, like most primates, they were also threatened by habitat destruction and fragmentation [73,74,75]. For one of these species, the white-headed marmoset, it is important to note that 195 roadkill incidents were reported between 2001 and 2015 in a single region of Brazil [15]. Given the exponential rate of habitat loss and land conversion in Brazil, it is possible that WVCs may pose a serious threat to local populations. Even a low risk can have an adverse effect on a population, and population declines caused by road mortality were seen in species such as the European common toad (*Bufo bufo*) and koala (*Phascolarctos cinereus*) [76,77]. Whilst it is reassuring that at-risk species appear less within the data, the loss of a single individual due to WVCs is an added pressure to already threatened populations, which is a conservation concern [11,78].

### 4.3. Countries, Hotspots and Coldspots

The countries with the most primate roadkill incidents were Kenya (35.8%), Brazil (27.5%), and South Africa (10.6%). This may be in part due to organizations within these countries having long-term projects collecting roadkill data. For example, Colobus Conservation in Diani Beach, Kenya, monitored local roadkill since 1998 and represents 937 incidents within the GPRD [79,80]. Similarly, most incidents involving marmosets in Brazil came from just three sources, an environmental consultancy agency (ViaFAUNA), a highway administration agency (BR-040), and a local roadkill study [81].

There are 88 primate range countries, and yet for over half, there were no reports of primate roadkill found. Noticeable primate-rich regions from where we did not receive any primate roadkill reports, or very few (i.e., coldspots), were Venezuela and the Guianas, western Africa and the Congo Basin, Madagascar, and mainland Southeast Asia and southern China. This absence of data does not indicate that primates are not involved in WVCs or even roadkill, rather it is simply that no records were obtained or reported there. Roadkill can be underreported due to reasons such as the dead animals simply being removed before it can be recorded, by humans or scavenging animals [13].

### 4.4. Road Expansion

With the prediction of 25 million kilometers of new road construction by 2050, we are undoubtedly in a time of unprecedented linear infrastructure expansion [7]. Ninety percent of these roads will be within developing nations that support much of the tropical and sub-tropical habitats where many primates live [82], including countries that harbor large numbers of primate species [6,83]. In the Amazon alone, there are 75 ongoing construction projects that will total 12 thousand kilometers of new roads [12,84]. With more roads in Brazil, roadkill is likely to become a greater threat to the country’s vertebrates. We acknowledge that rapid infrastructure expansion is essential to facilitate development; however, the uncontrolled scale of road building in Brazil will have considerable adverse effects on the environment [85,86,87].

The earth’s terrestrial surface is divided into more than 600,000 patches due to roads, and valuable roadless patches remain largely unprotected [87]. Of the remaining patches, more than half of them are less than one square kilometer, and only 7% are greater than 100 km^2^; thus, we are faced with questioning whether highly sensitive species will be able to persist in such small patches [88]. As the remaining valuable roadless areas are further divided, vulnerable primate species (and all wildlife) will be faced with inhabiting areas near roads. With rapid anthropogenic changes, it is likely that species currently not reported in the GPRD may be included in the near future. In recent years, camera trap evidence revealed that critically endangered Bornean orangutans (*Pongo pygmaeus*) occasionally travel on the ground, with individuals using logging roads [89,90]. While it is reassuring that Bornean orangutans are showing some adaptive responses to anthropogenic habitat disturbance, ultimately, they are at an increased risk of WVCs if they increase regular use of linear infrastructures.

### 4.5. Mitigating Primate Roadkill

Various strategies for mitigating WVCs are implemented across the globe and may include wildlife underpasses and bridges, fences, speed bumps to reduce traffic speed, and signage to warn drivers [91,92,93,94]. The effectiveness of a mitigation measure depends on a combination of variables, including the location, habitat type, road type, target species, and whether multiple strategies are implemented simultaneously [95]. To ensure the success of a mitigation measure, it is advised that data collection be carried out to determine how and why WVCs are occurring in a particular area, which will then inform the most effective strategy to implement, and then continued monitoring must be carried out to assess the effectiveness of the measure/strategy [95,96,97]. An example of a common primate WVC mitigation measure is a canopy bridge, which are often implemented in primate roadkill hotspots such as Kenya and Brazil [98,99]. Increasing canopy connectivity between habitats fragmented by roads was shown to reduce the risk of WVC and mortality for arboreal primates, and the number of publications sharing information about the design, implementation, and success of canopy bridges is increasing [100,101,102].

Likewise, the effectiveness of road signs increases with amongst others its universality (i.e., using one shape, one set of colors and one, generalized animal depiction: much of this is coded by the 1968 Vienna Convention on Road Signs and Signals), local adaptations (for instance, background color to reduce glare), and the type of animal that is featured on the sign [103]. During our research, we came across a good number of local, well recognized, and charismatic primates that feature on road warning signs, including macaques in Japan, Indonesia and Gibraltar, langurs in Sri Lanka, baboons in South Africa, gibbons in India, and howlers in Colombia. These signs were either diamond-shaped with a yellow background or triangular white with a red rim, and the primates that were depicted were universally black with relatively few distinguishing features. While there is a danger that depicting too many different species may lower instant recognition for drivers, we assume that along especially for the secondary roads depicting local species (rather than a universal generic monkey) increases the likelihood of drivers taking notice.

Though the global primate roadkill database is a valuable tool for primate conservation, it is clear that the issues leading to primate roadkill differ dramatically across the globe. It is important to note that previous incidents of roadkill resulting in apparent ‘hotspots’ may have already reduced the population size of the target species; thus, those ‘hotspots’ may not always be indicative of the best locations for potential mitigation measures [18]. To properly quantify and reduce WVCs and wildlife roadkill, more research needs to be conducted at a local level. There are hopeful signs that governments are starting to take notice of these issues, demonstrated in Costa Rica as they are in the process of adopting legislation which will make it a requirement to connect fragmented habitat with canopy bridges where new roads are constructed [104].

## 5. Conclusions

In summary, the GPRD is a work in progress, but to our knowledge, it already represents the most comprehensive data repository for global information on primate roadkill. Our general data analysis identified species most at risk; however, we acknowledge that there are still large gaps within the data. The database currently reports at least 107 primate species in roadkill incidents, though it is highly likely that many more primate species are at risk.

This database will be useful for future scientific research and will remain available for both contributions and used to advance our understanding of primate roadkill incidents. We strongly advocate for collaboration between researchers and policy makers to find effective solutions to mitigate the impacts WVCs have on primates. Ultimately, we hope that this database will be a valuable tool for primate conservation.

The GPRD website, and the database itself, including guidance on how to use it, are available to view here: https://gprd.mystrikingly.com/ (accessed on 4 April 2023).

## Figures and Tables

**Table 1:** Number of individual primates and species reported as roadkill in 41 countries.

Continent	Country	Species	Individuals	Total
Neotropics	Argentina	2	22	
	Barbados	1	1	
	Brazil	26	788	
	Colombia	4	4	
	Costa Rica	4	41	
	Ecuador	5	12	
	Guatemala	1	1	
	Mexico	3	55	
	Nicaragua	1	1	
	Panama	2	4	
	Paraguay	1	4	
	Peru	2	7	940
Asia	Bangladesh	5	24	
	Cambodia	1	3	
	China (incl. Hong Kong)	3	88	
	India	13	179	
	Indonesia	9	20	
	Japan	1	23	
	Malaysia	5	39	
	Nepal	1	1	
	Saudi Arabia	1	1	
	Singapore	3	10	
	Sri Lanka	3	3	
	Taiwan	1	37	
	Thailand	2	5	
	Vietnam	2	2	435
Africa	Democratic Republic of Congo	1	1	
	Ethiopia	3	18	
	Equatorial Guinea	1	1	
	Gabon	1	1	
	Kenya	6	1,026	
	Madagascar	6	12	
	Malawi	1	2	
	Morocco	1	6	
	Namibia	1	2	
	Rwanda	3	4	
	Senegal	1	1	
	South Africa	5	302	
	Tanzania	6	66	
	Uganda	12	37	
	Zambia	3	8	1487
All				2862

**Table 2:** Global IUCN Red List status of primates reported as roadkill in GPRD (only including primates identified to species level).

IUCN Red List Categories	Individuals	Species
Not at risk		
Least Concern	1807	37
Near-Threatened	61	11
Total	1868	48
At risk		
Vulnerable	357	22
Endangered	222	27
Critically Endangered	16	8
Total	595	57

## Data Availability

All data used are available at https://gprd.mystrikingly.com/ (accessed on 4 April 2023).
